# Posttraumatic stress in newly diagnosed epilepsy: associations with early maladaptive schemas, anxiety, and quality of life in a 6-month prospective study

**DOI:** 10.55730/1300-0144.6345

**Published:** 2026-05-31

**Authors:** Mehmet ÖZTÜRK, Pınar ÖZTÜRK, Fügen SÖNMEZ ÇETİNKAYA, İlker ÖZDEMİR

**Affiliations:** 1 Department of Psychiatry, Dr. Abdurrahman Yurtaslan Ankara Oncology Training and Research Hospital, Ankara, Turkiye; 2 Department of Neurology, Yenimahalle Training and Research Hospital, Ankara Yıldırım Beyazıt University, Ankara, Turkiye; 3 Department of Psychiatry, Bursa City Hospital, Bursa, Turkiye

**Keywords:** Epilepsy, posttraumatic stress disorder, anxiety, cognitive schemas, quality of life

## Abstract

**Background/aim:**

Epilepsy is a chronic neurological disorder that significantly affects psychosocial functioning, self-perception, and quality of life. Emerging evidence suggests that posttraumatic stress (PTS) symptoms may be more prevalent in individuals with epilepsy than previously recognized, particularly following seizure-related experiences perceived as traumatic. The aim of this study was to evaluate short- and long-term PTS levels in individuals with newly diagnosed epilepsy and examine their associations with anxiety, depression, early maladaptive schemas, and quality of life.

**Materials and methods:**

In this prospective study, 31 individuals with newly diagnosed epilepsy were enrolled. Participants completed a sociodemographic data form, the Post-Traumatic Stress Disorder Checklist for DSM-5, the Young Schema Questionnaire–Short Form 3, the Beck Anxiety Inventory, the Beck Depression Inventory, and the Quality of Life in Epilepsy Inventory. Statistical analyses were performed with significance set at p < 0.05.

**Results:**

Anxiety showed the strongest association with PTS symptoms at both the first- and sixth-month assessments. The Enmeshment/Dependence schema was also significantly associated with higher PTS levels, and increased total schema scores correlated with greater traumatic stress. Female sex was associated with higher PTS scores. Quality of life showed significant declines, particularly in cognitive functioning, overall health status, and overall quality of life, and these declines were negatively correlated with PTS symptom severity.

**Conclusion:**

PTS symptoms in newly diagnosed epilepsy are closely associated with anxiety, early maladaptive cognitive schemas, and impaired quality of life. These findings emphasize the importance of incorporating trauma-informed psychiatric assessment and early psychological interventions into routine epilepsy care, in addition to seizure management.

## 1. Introduction

Epilepsy is a chronic neurological disorder characterized by recurrent, unpredictable, and sudden disruptions in normal brain activity, referred to as epileptic seizures [1]. Generalized tonic-clonic seizures (GTCSs), formerly referred to as grand mal seizures, constitute one of the most common seizure types in epilepsy [2]. These seizures typically begin with a tonic phase, followed by clonic muscle contractions, and are often perceived as frightening by both patients and their relatives [3]. Beyond their clinical presentation, GTCSs impose substantial demands on daily life, including the need for continuous medication adherence, regular medical follow-up, and preparedness for acute medical emergencies, thereby adversely affecting quality of life [3]. In addition, GTCSs are frequently accompanied by sudden loss of consciousness, loss of bodily control, risk of physical injury, and distressing postictal experiences, all of which may contribute to the perception of seizures as potentially traumatic events [4]. The unpredictable nature of seizures, their tendency to recur, the risk of accidents and physical injuries, and the associated fear of death collectively contribute to the potentially traumatizing impact of these events [5,6].

Psychological trauma typically arises from extraordinary and unexpected events that involve a perceived threat of death or injury to oneself or others, accompanied by intense fear, horror, and helplessness [7]. Although trauma most commonly follows events such as natural disasters, wars, or accidents, it may also arise in the context of severe medical conditions. Posttraumatic stress disorder (PTSD) is a major psychiatric condition that can emerge months or even years after exposure to traumatic events and is characterized by symptoms of reexperiencing, avoidance of trauma-related cues, and persistent negative alterations in mood, cognition, and hyperarousal [8]. The lifetime prevalence of PTSD has been estimated at approximately 8.3% [9]. Importantly, subclinical posttraumatic stress (PTS) symptoms are considerably more common among individuals exposed to traumatic events than full diagnostic PTSD [10]. Although anxiety and depression have been extensively examined in epilepsy, traumatic stress symptoms remain relatively underexplored in this population [5,11]. Furthermore, the relationship between PTS symptoms and early maladaptive schemas in individuals with epilepsy has received limited attention in the existing literature. Early maladaptive schemas may influence how seizure-related threat is appraised, encoded, and later recalled.

Schemas are enduring cognitive structures involving emotions, memories, beliefs, and broad patterns of information processing that guide behavior. They serve as core cognitive structures that shape individuals’ understandings of themselves, others, and their experiences [12]. Schemas develop during early childhood and gradually consolidate through life experiences. Individual characteristics and cultural factors may also play important roles in the development and activation of schemas [13].

In PTSD, impairments across multiple domains of functioning are accompanied by prominent cognitive difficulties, including problems in memory, attention, concentration, and behavior. These cognitive difficulties have been linked to the presence of maladaptive schemas, which are commonly observed in PTSD, as in many other psychiatric disorders [14]. Enmeshment/dependence, defectiveness, and failure schemas have been consistently associated with the severity of PTS symptoms [15].

This study aimed to evaluate PTS levels in individuals diagnosed with epilepsy presenting with GTCSs at the first and sixth months following the initial seizure and to examine the associations between changes in PTS levels, early maladaptive schemas, and quality of life. A secondary aim was to investigate the relationships between PTS levels and sociodemographic characteristics, including sex, age, marital status, and years of education, as well as clinical variables such as anxiety and depression.

We hypothesized that increases in PTS symptoms and maladaptive schema levels from the early to the later period following the first seizure would be associated with reductions in quality of life; that PTS levels at both time points would be positively associated with anxiety, depression, and maladaptive schemas and negatively associated with quality of life; and that sociodemographic factors, including female sex and the presence of physical injury during the first seizure, would be positively associated with PTS levels.

## 2. Materials and methods

### 2.1. Selection of participants and procedure

Between May 2022 and May 2023, we enrolled 50 individuals who presented to the Neurology Outpatient Clinic of Ankara Yıldırım Beyazıt University Yenimahalle Training and Research Hospital after a first GTCS and were subsequently diagnosed with epilepsy by a neurologist. Seizures were classified according to the 2017 International League Against Epilepsy operational classification, and only generalized-onset tonic-clonic seizures were included in order to maintain a clinically more homogeneous sample regarding seizure phenomenology and potential trauma-related burden. Inclusion criteria were being between 18 and 65 years of age, having a first-time diagnosis of epilepsy presenting with GTCS, and being literate. Exclusion criteria included focal-onset seizures evolving to bilateral tonic-clonic seizures, a previous diagnosis of epilepsy or a clinician-confirmed history of epileptic seizures, the presence of an ongoing neurodevelopmental disorder, alcohol and/or substance use disorder, a known history of PTSD, and current psychotic disorders or bipolar disorder. The required sample size was determined using a priori power analysis conducted with G*Power software (Heinrich Heine University Düsseldorf, Düsseldorf, Germany). Based on a type I error rate of 0.05, statistical power of 0.80, and effect size of 0.87, the minimum required sample size was calculated as 28 participants [6]. Given the prospective design of the study and the anticipated risk of loss to follow-up, recruitment was continued until 50 participants were enrolled. After obtaining written informed consent, neurological and psychiatric evaluations were conducted by the same independent neurologist and psychiatrist to minimize assessment bias. Clinical assessment scales were administered once between days 30 and 35 after the first seizure (first-month assessment window) and were repeated at the sixth month. Because psychometric assessments were first administered during the first-month assessment window, participants who did not attend this visit (n = 8) had no baseline psychometric data available. In addition, 11 participants who did not attend the sixth-month follow-up were lost to follow-up and therefore not included in the final analyses, resulting in a final sample of 31 participants. A flow diagram illustrating participant recruitment, follow-up, and inclusion in the final analyses is presented in the Figure.

This was a prospective hospital-based observational clinical study with no investigator intervention. Ethical approval was obtained from the local ethics committee on 27 April 2022 (Approval No: E-2022-21). The study was conducted in accordance with the 1964 Declaration of Helsinki and its later amendments.

### 2.2. Data evaluation tools

#### 2.2.1. Sociodemographic data form

A sociodemographic data form was developed by the authors to collect information regarding participants’ sociodemographic and clinical characteristics, as well as their personal and family histories.

#### 2.2.2. Beck Depression Inventory (BDI)

The BDI is a 21-item self-report scale designed to assess emotional, cognitive, somatic, and motivational symptoms of depression [16]. The Turkish validity and reliability study of the scale was conducted by Hisli [17]. Higher scores indicate greater severity of depressive symptoms.

#### 2.2.3. Beck Anxiety Inventory (BAI)

The BAI is a 21-item self-report scale developed by Beck et al. to assess the severity of anxiety symptoms [18]. The Turkish validity and reliability study of the scale was conducted by Ulusoy [19]. Higher scores indicate greater severity of anxiety symptoms.

#### 2.2.4. Young Schema Questionnaire–Short Form 3 (YSQ-SF3)

The YSQ-SF3 consists of 90 items rated on a 6-point Likert-type scale and was originally developed to assess 18 early maladaptive schemas [13]. In the Turkish adaptation conducted by Soygüt et al. [20], schema domains were combined and evaluated within 14 maladaptive schema dimensions, which were used in the present study.

#### 2.2.5. Post-Traumatic Stress Disorder Checklist for DSM-5 (PCL-5)

The PCL-5 was developed to assess the severity of PTS symptoms and does not establish a clinical diagnosis of PTSD. It consists of 20 items across four symptom clusters [21]. The Turkish adaptation of the scale was conducted by Boysan et al. and a cut-off score of 48 was determined [22].

#### 2.2.6. Quality of Life in Epilepsy Inventory (QOLIE-31)

The QOLIE-31 is a disease-specific scale developed to assess quality of life in individuals with epilepsy and it consists of 31 items across seven subscales [23]. The Turkish validity and reliability study of the scale was conducted by Mollaoğlu et al. [24].

### 2.3. Statistical analysis

Statistical analyses were performed using IBM SPSS Statistics 26 for Windows (IBM Corp., Armonk, NY, USA). Categorical variables were summarized as frequencies and percentages, whereas continuous variables were presented as means ± standard deviations. The normality of continuous variables was assessed using the Shapiro–Wilk test, given the sample size of fewer than 50 participants. Comparisons of scale scores between the first- and sixth-month assessments were performed using paired-samples t-tests for normally distributed data and the Wilcoxon signed-rank test for nonnormally distributed data. For clarity of presentation, these results were reported in separate tables according to the statistical method used. Associations among the applied scale scores were examined using Spearman correlation analysis. False discovery rate (FDR) correction was performed using the Benjamini–Hochberg procedure and adjusted q-values are reported. Correlations with values of q < 0.05 were considered statistically significant after correction. To examine variables associated with PTS levels, we performed exploratory multiple linear regression analysis using a stepwise variable selection approach, given the modest sample size of the study. Because of the relatively small sample and the exploratory design, regression findings were interpreted cautiously due to the potential risk of model instability and overfitting. Independent variables that showed significant correlations with PTS levels, along with relevant sociodemographic variables, were entered into the model. The 95% confidence intervals were calculated and reported for regression coefficients. Statistical significance was set at p < 0.05.

## 3. Results

A total of 50 individuals with newly diagnosed epilepsy presenting with GTCSs were initially enrolled, and 31 participants who completed the sixth-month follow-up were included in the final analyses. The mean age of the sample was 39.0 ± 16.3 years and 61.3% (n = 19) of the participants were female. More than half of the participants were married (54.8%) and 45.2% reported having children. The mean duration of education was 11.6 ± 2.9 years and 64.5% of the participants were unemployed at the time of assessment. Only 12.9% were living alone. A history of psychiatric comorbidity was reported by 6.5%, and 29.0% had an additional medical comorbidity. Nearly half of the sample (48.4%) experienced physical trauma during their first seizure, whereas status epilepticus at the first seizure was observed in only one participant (3.2%). During the 6-month follow-up period, 35.5% of the participants remained seizure-free and 77.4% reported regular use of antiepileptic medication. According to the Turkish PCL-5 cut-off score of 48, 3 participants (9.6%) at month 1 and 5 participants (16.1%) at month 6 met the threshold for probable PTSD.

When the first-month and sixth-month scale scores of the participants were compared, a statistically significant difference was observed in BDI scores, with a moderate effect size (Z = −2.798, p = 0.005, r = 0.50). Examination of changes in QOLIE-31 scores revealed statistically significant changes with moderate effect sizes in the QOLIE-31 Cognitive Function (t = 2.861, p = 0.008, Cohen d = 0.51) and QOLIE-31 Overall Quality of Life (t = 2.843, p = 0.008, Cohen d = 0.51) subscales. In contrast, changes in QOLIE-31 Overall Health Status (t = 4.997, p < 0.001, Cohen d = 0.90) and QOLIE-31 Total Score (t = 4.768, p < 0.001, Cohen d = 0.86) were statistically significant with large effect sizes. Changes in PTS levels were also statistically significant and demonstrated a moderate effect size (t = −2.875, p = 0.007, Cohen d = 0.52). Regarding changes in YSQ-SF3 subscales, statistically significant differences with moderate effect sizes were observed in Self-Sacrifice (t = −3.529, p = 0.001, Cohen d = 0.63), Entitlement/Insufficient Self-Control (Z = −2.736, p = 0.006, r = 0.49), Abandonment (Z = −2.356, p = 0.018, r = 0.42), and Unrelenting Standards (Z = −1.990, p = 0.047, r = 0.36). The change in the YSQ-SF3 Total Score was statistically significant with a large effect size (Z = −2.870, p = 0.004, r = 0.52) (Tables 1 and 2).

At the first-month assessment, correlations between PTS levels and age, as well as other scale scores, were examined. Although PTS levels showed positive correlations with BAI scores and the YSQ-SF3 Enmeshment/Dependence subscale and negative correlations with QOLIE-31 Seizure Anxiety, QOLIE-31 General Health Status, and QOLIE-31 Total Score, these correlations lost their significance after applying FDR correction (Table 3). At the sixth-month assessment, PTS levels showed significant positive correlations with BDI and BAI scores and significant negative correlations with age, QOLIE-31 Overall Health Status, and QOLIE-31 Total Score. Although PTS levels showed positive correlations with the YSQ-SF3 Abandonment subscale and negative correlations with QOLIE-31 Seizure Worry and QOLIE-31 Cognitive Function, these correlations lost their significance after applying FDR correction (Table 3).

In exploratory stepwise multiple linear regression analysis, female sex, BAI scores, presence of physical trauma during the first seizure, and YSQ-SF3 Enmeshment/Dependence scores showed independent associations with PTS levels at the end of the first month. The overall model was statistically significant (F (4, 26) = 10.449, p < 0.001) and explained a substantial proportion of the variance in PTS levels (R^2^ = 0.79; adjusted R^2^ = 0.56). Sex was a significant contributor to the model (B = −11.726, β = −0.366, p = 0.006), indicating higher PTS scores in women compared to men. The presence of physical trauma during the first seizure was also significantly associated with higher PTS levels (B = −8.616, β = −0.276, p = 0.037). Anxiety severity was positively associated with PTS levels (B = 0.447, β = 0.348, p = 0.011) and the YSQ-SF3 Enmeshment/Dependence subscale was also positively associated with PTS levels (B = 0.772, β = 0.424, p = 0.002) (Table 4). Other sociodemographic variables, BDI scores, other YSQ-SF3 subscales, and all QOLIE-31 subscales were excluded from the model due to a lack of significant contribution.

In exploratory stepwise multiple linear regression analysis, BAI score was the only variable retained as independently associated with PTS levels at the sixth month (F (1, 29) = 25.176, p < 0.001), explaining a substantial proportion of variance in PTS scores, although the adjusted R^2^ value was considerably lower (R^2^ = 0.68; adjusted R^2^ = 0.45). Each one-point increase in anxiety score was associated with an average increase of 0.85 points in PTS score (B = 0.856, β = 0.682, p < 0.001) (Table 4). Sociodemographic variables, BDI scores, all YSQ-SF3 subscales, and all QOLIE-31 subscales were not retained in the final model because they did not provide a significant incremental contribution.

## 4. Discussion

In this study, changes in PTS levels over time were examined in individuals with newly diagnosed epilepsy presenting with GTCSs, together with the potential roles of early maladaptive schemas and quality of life. The findings indicate that epilepsy is associated with substantial emotional stress from the initial phases, accompanied by a significant increase in PTS levels over time. In the early period, anxiety level, female sex, presence of physical trauma during the first seizure, and the Enmeshment/Dependence schema were significantly associated with PTS levels, whereas in the later period, anxiety level remained the primary factor associated with traumatic stress.

In the present study, PTS levels showed a significant increase at the sixth month compared to the first month. Soncin et al. reported that PTS symptoms in patients with epilepsy are often overlooked due to the unpredictable nature of seizures and the associated sense of loss of control; however, a substantial proportion of patients develop clinically significant traumatic responses [6]. Another study emphasized that epileptic seizures may evoke intense fear of death, helplessness, and perceived loss of control [25]. These findings suggest that an epilepsy diagnosis should be considered not only as a neurological condition but also as a potential source of psychological trauma. The progressive increase in traumatic stress symptoms over time may be related to the risk of seizure recurrence, social restriction, fear of stigma, and the persistence of perceived loss of control. The ongoing uncertainty associated with epilepsy and anticipatory anxiety regarding possible future seizures may further contribute to the persistence and progression of traumatic stress symptoms over time. Additionally, physical injury experienced during seizures may contribute to the consolidation of traumatic memories [26].

In line with the present findings, the significant increase in depressive symptom levels observed alongside PTS levels at the sixth month, as well as the significant positive correlation between these two variables, highlights the close relationship between traumatic stress and depressive symptoms. This finding suggests that the chronicity of PTS symptoms may increase the likelihood of developing depressive symptoms over time, potentially through trauma-related emotional exhaustion, hopelessness, and feelings of worthlessness. A high rate of comorbidity between PTSD and depression has been consistently reported in the literature [27]. Previous studies have suggested that shared stress-related neurobiological mechanisms may contribute to the association between traumatic stress and depressive symptoms [28]. Additionally, the presence of early maladaptive schemas may increase vulnerability to depression and facilitate the development of depressive symptoms secondary to traumatic experiences [13]. Therefore, in individuals diagnosed with epilepsy, it is important to monitor depressive symptoms alongside PTS symptoms and to implement comprehensive psychosocial interventions.

Anxiety emerged as a central factor in relation to traumatic stress throughout the course of the illness. Clinical levels of anxiety symptoms have been reported in approximately 15%–50% of patients with epilepsy [29]. Anxiety disorders in epilepsy have been associated with multiple factors, including seizure frequency, the unpredictable nature of the disease, medication-related side effects, social stigma, and fear of loss of control during seizures [30].

Anxiety is closely linked to the reexperiencing and hyperarousal components of traumatic stress. Elevated anxiety levels may intensify threat perception, thereby strengthening the sensory and cognitive traces of traumatic experiences [15]. In individuals with epilepsy, this mechanism may operate in a similar manner, as anticipatory anxiety related to seizure occurrence and fear of loss of control can continuously activate traumatic memories. Accordingly, anxiety may play a mediating role in the maintenance of trauma and the chronicity of PTS symptoms. From a neurobiological perspective, shared pathophysiological mechanisms between epilepsy and anxiety may further support this association. Although not directly assessed in the present study, stress-related neurobiological mechanisms may partially contribute to the relationship between anxiety and traumatic stress responses in epilepsy [31]. Elevated anxiety levels may reinforce the reexperiencing and hyperarousal components of traumatic stress [32].

In this context, anxiety in individuals diagnosed with epilepsy should be considered not merely as an accompanying symptom but as a central factor that may trigger and sustain traumatic stress responses. Furthermore, the strong association observed between BAI and PCL-5 scores may partly reflect shared method variance and overlapping anxiety-related self-report content between the two instruments rather than entirely distinct psychopathological constructs. In clinical practice, early assessment of anxiety levels in individuals with newly diagnosed epilepsy may be of critical importance in preventing the progressive course of traumatic stress. This perspective is consistent with previous studies demonstrating that targeting anxiety through trauma-focused cognitive behavioral therapies is associated with significant reductions in PTS symptoms [33].

Female sex has been consistently identified as an important risk factor for the development of PTS symptoms. The likelihood of developing PTS symptoms has been reported to be approximately twofold higher in women compared to men [34]. Following traumatic exposure, women are more likely to exhibit dissociative responses, difficulties in emotional processing, and increased tendencies toward self-blame [35]. In addition, studies have shown that female patients may experience greater psychosocial burden following an epilepsy diagnosis [36]. These stressors may include loss of social roles, concerns related to motherhood, and heightened perceptions of stigma, all of which may contribute to increased vulnerability to PTS symptoms. Therefore, the effect of female sex on PTS symptoms is a multilayered process, involving not only biological but also psychosocial and social aspects. From a clinical perspective, early screening for PTS symptoms, together with anxiety and depression, may be particularly important in female individuals newly diagnosed with epilepsy to identify individuals at increased risk.

Physical injury during a seizure, such as falling, head trauma, or the experience of a life-threatening loss of control, may reinforce the traumatic nature of the event [37]. In this context, experiencing physical trauma during the first epileptic seizure may strengthen the perception of a threat to bodily integrity, thereby facilitating the emergence of PTS symptoms. The literature suggests that postepilepsy PTS symptoms are often related to the subjective experience of seizures as traumatic events. Previous studies have reported that the incidence of PTS symptoms in such individuals is significantly higher than in the general epilepsy population and that factors including prior trauma exposure, dissociative tendencies, and elevated anxiety levels play an important role in this process [6]. Accordingly, the presence of physical trauma during the first seizure may increase the likelihood that the seizure is perceived as threatening and experienced as a traumatic event. Although direct evidence regarding the impact of physical injury during the first seizure on PTS levels remains limited, current findings suggest that trauma perception in epilepsy is multidimensional. Physical injury may represent one of the key contributors to the development of traumatic stress responses. This finding of the present study contributes to a relatively underexplored area of the literature and highlights the need for further research in this field.

With respect to early maladaptive schemas, the Enmeshment/Dependence schema emerged as an independent factor associated with PTS symptom severity in the exploratory regression model at the first-month assessment. Although the positive correlation observed at the sixth month did not reach statistical significance, the early-phase finding is consistent with previous studies suggesting that maladaptive schema patterns may contribute to vulnerability to traumatic stress responses [14,15]. Individuals characterized by such schema patterns may have difficulty tolerating experiences of loss of control following traumatic events. From a schema-theoretical perspective, the Enmeshment/Dependence schema reflects difficulties in autonomous functioning and a pervasive belief that one cannot cope without close guidance and support from significant others [12,13]. In the context of newly diagnosed epilepsy, the sudden and uncontrollable nature of seizures, the possibility of losing consciousness, and the resulting need to rely on relatives or healthcare providers may be experienced as direct threats to perceived autonomy and self-sufficiency [14,15]. These illness-related experiences can reactivate Enmeshment/Dependence themes, intensify feelings of helplessness and dependence, and thereby amplify the subjective traumatic impact of seizure-related events [13–15,37]. This mechanism may help explain why the Enmeshment/Dependence schema in particular was associated with higher PTS symptoms in the early phase of the illness. In addition, the observed increase in overall schema scores in our study is consistent with cognitive models proposing that traumatic experiences activate preexisting cognitive schemas and contribute to the maintenance of psychopathology [12]. However, the interpretation of increased YSQ-SF3 scores over a 6-month period requires caution. Early maladaptive schemas are generally conceptualized as relatively stable cognitive structures that originate in childhood and persist across the lifespan [12,13]. Therefore, the observed increase may not necessarily reflect a true structural change in underlying schemas [13]. One possible explanation is that the YSQ-SF3 is sensitive to schema activation under conditions of ongoing psychological distress. In patients with newly diagnosed epilepsy, uncertainty regarding seizure recurrence, fear of loss of control, functional restrictions, and illness-related psychosocial burden may increase the salience of preexisting maladaptive schemas, leading to higher self-reported schema scores over time [3–5,12,36]. Alternatively, persistent exposure to illness-related stressors may gradually reinforce maladaptive cognitive beliefs and contribute to the consolidation of schema-related patterns [12,13,32]. Future longitudinal studies with longer follow-up periods and repeated assessments are needed to clarify whether increases in schema scores primarily reflect temporary schema activation or more enduring changes in maladaptive cognitive structures [6,26,37]. Therefore, it may be said that PTS symptoms related to epilepsy are also connected to cognitive biases at the schema level [12–14,32]. Epilepsy is a lifelong condition that may challenge an individual’s sense of control. In this context, the reactivation of early maladaptive schemas, particularly those related to enmeshment/dependence, failure, and self-sacrifice, may contribute to the development of PTS symptoms. From a clinical perspective, attention to individual cognitive characteristics may be important for both the diagnostic evaluation and therapeutic planning of PTS symptoms in patients with epilepsy [12,13,30,33].

A significant decline in quality of life was observed among individuals with epilepsy, particularly in the domains of cognitive functioning, overall health status, and overall quality of life. Mollaoğlu et al. demonstrated that quality of life in individuals with epilepsy deteriorates most prominently in cognitive performance, energy level, and social functioning and that this deterioration is more strongly associated with stigma, anxiety, and depression than with seizure frequency or medication-related side effects [24]. The negative correlation between quality of life and PTS symptoms suggests that traumatic stress may adversely affect psychological well-being. It may also negatively influence cognitive and somatic functioning. This finding indicates that reductions in quality of life among individuals with epilepsy are also shaped by psychological factors. Furthermore, previous research has shown that depression and anxiety present strong associations with quality of life in epilepsy, whereas epilepsy-specific variables such as seizure type and frequency exert relatively weaker effects [38]. These findings support the association observed in our study between PTS levels and quality of life. Elevated PTS symptoms may further increase anxiety levels accompanying epilepsy, thereby negatively influencing disease management and overall quality of life.

One of the main strengths of this study is its prospective design, which allowed for the monitoring of PTS symptoms over a 6-month period in individuals with newly diagnosed epilepsy. In addition, this study is among the few prospective studies to evaluate trauma-related stress, anxiety, depression, quality of life, and early maladaptive schemas within a comprehensive framework in individuals with epilepsy. Given the limited literature on schema-related processes in epilepsy, these findings may contribute to a better understanding of the psychological mechanisms associated with traumatic stress responses in this population. However, several limitations should be considered when interpreting the findings. First, the single-center design, relatively small sample size, and absence of a healthy control group or a comparison group involving another newly diagnosed chronic medical condition limit the generalizability of the results. It remains unclear whether the observed increases in traumatic stress symptoms and schema scores are specific to epilepsy and seizure experiences or reflect a broader psychological response to chronic illness. In addition, reliance on self-report measures may have increased the risk of subjective response bias. Potentially relevant clinical variables, including seizure frequency, seizure severity, and antiepileptic medication characteristics, were not systematically evaluated and therefore could not be included in the analyses. Furthermore, the regression analyses should be interpreted cautiously because of the modest sample size, the exploratory nature of the models, the relatively large discrepancies between R^2^ and adjusted R^2^ values, and the use of a stepwise variable selection approach, all of which may increase the risk of model instability and overfitting. In addition, the strong association observed between BAI and PCL-5 scores may partly reflect shared method variance and overlapping anxiety-related self-report content between the two instruments rather than entirely distinct psychopathological constructs. Although FDR correction was applied to reduce the risk of type I error in the correlation analyses, the relatively large number of statistical comparisons may still have increased the possibility of spurious associations. Another limitation of the present study was the relatively high attrition rate during follow-up. Because psychometric assessments were first administered during the first-month evaluation, no baseline scale data were available for participants who did not attend this visit. In addition, incomplete follow-up data limited formal comparisons between completers and noncompleters and prevented a reliable characterization of the missing data mechanism. Therefore, the possibility that individuals with greater psychological distress or higher PTS symptom severity were less likely to continue follow-up cannot be excluded, potentially resulting in an underestimation of traumatic stress levels in the study population. Despite these limitations, the present findings provide preliminary prospective data regarding traumatic stress responses in individuals with newly diagnosed epilepsy. Future longitudinal studies including larger samples, longer follow-up periods, healthy controls, and comparison groups involving other chronic medical conditions are needed to clarify the causal and longitudinal relationships among traumatic stress symptoms, maladaptive schemas, anxiety, and quality of life in epilepsy.

This study demonstrates that PTS symptoms may increase over time in individuals with newly diagnosed epilepsy and that this increase may be associated with anxiety levels, certain early maladaptive schemas, and deterioration in quality of life. Anxiety showed the strongest and most consistent association with traumatic stress severity in both the early and late phases, highlighting the importance of early psychiatric assessment following an epilepsy diagnosis. In the early phase, female sex and exposure to physical trauma during the first seizure were associated with higher PTS symptom levels, suggesting that these factors may identify individuals who could require closer clinical monitoring.

Overall, the findings of this study indicate that epilepsy is not merely a neurological condition; it is a life-altering event that may confer psychiatric vulnerability. Elevated anxiety levels and the activation of early maladaptive schemas appear to play a key role in maintaining traumatic stress responses. Accordingly, integrating trauma-focused and early supportive interventions, particularly cognitive-behavioral and schema-based approaches, into the treatment process of individuals with epilepsy may be clinically beneficial. Greater attention to psychological symptoms and quality of life may also contribute to improved patient well-being and long-term functioning. Given the relatively small sample size and limited follow-up period, further multicenter and comparative studies with larger samples and longer follow-up durations are warranted to better clarify traumatic stress responses associated with epilepsy.

## Figures and Tables

**Figure:**
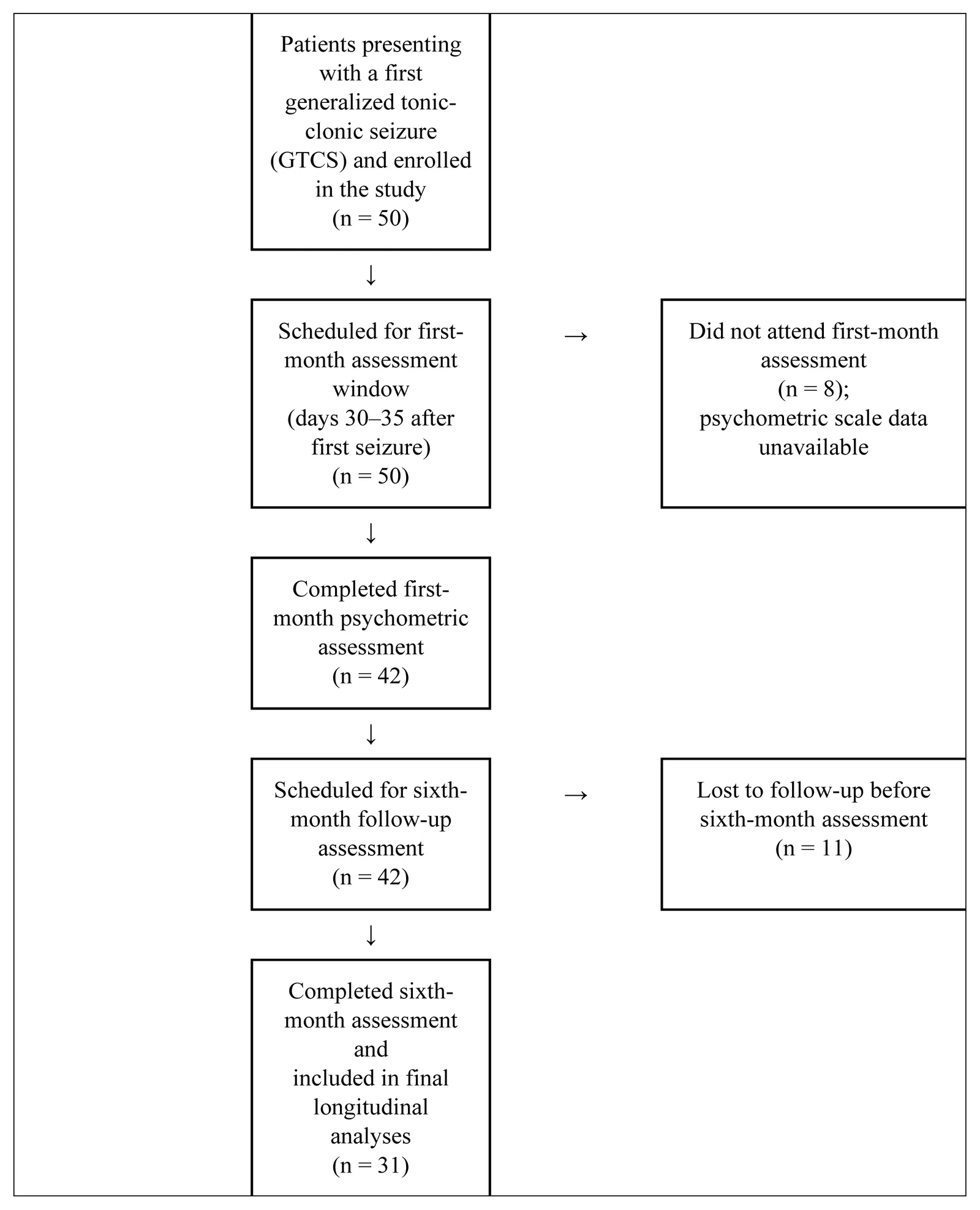
Flow diagram of participant recruitment and follow-up process.

**Table 1:** Scale scores for first month and sixth month and comparisons.

	Scale	Month 1 scores (mean±SD)	Month 6 scores (mean±SD)	t	df	p	Cohen’s d
BAI		17.97±12.37	20.16±12.02	−1.744	30	0.091*	0.31
QOLIE-31	Social Function	18.74±3.74	18.35±3.28	0.935	30	0.357*	0.17
	Cognitive Function	21.45±4.46	20.13±3.80	2.861	30	0.008*	0.51
	Medication Effect	7.87±2.91	8.55±1.93	−1.366	30	0.182*	0.25
	Overall Quality of Life	8.90±1.74	8.10±1.66	2.843	30	0.008*	0.51
	Overall Health Status	63.87±13.08	52.58±14.83	4.997	30	<0.001*	0.90
	Total	168.52±20.26	153.52±20.93	4.768	30	<0.001*	0.86
PCL-5		24.68±15.86	34.35±15.09	−2.875	30	0.007*	0.52
YSQ-SF3	Emotional Deprivation	9.68±4.08	10.26±4.72	−1.448	30	0.158*	0.26
	Self-Sacrifice	14.68±5.29	16.03±5.61	−3.529	30	0.001*	0.63

BAI: Beck Anxiety Inventory; QOLIE-31: Quality of Life in Epilepsy Inventory; PCL-5: Post-Traumatic Stress Disorder Checklist for DSM-5; YSQ-SF3: Young Schema Questionnaire–Short Form 3; sd: standard deviation.

* Variables meeting normal distribution assumptions and analyzed using paired-samples t-tests are presented.

**Table 2:** Scale scores for first month and sixth month and comparisons.

	Scale	Month 1 (median-IQR)	Month 6 (median-IQR)	Z	p	Magnitude (r)
BDI		17.35 (15–11)	19.00 (17–15)	−2.798	0.005*	0.50
QOLIE-31	Seizure Worry	14.23 (15–7)	13.35 (14–4)	−1.534	0.125*	0.28
	Emotional Wellbeing	18.48 (18–4)	18.16 (18–2)	−0.576	0.565*	0.10
	Energy Fatigue	14.97 (15–5)	14.29 (14–2)	−1.148	0.251*	0.21
YSQ-SF3	Failure	12.35 (10–7)	11.61 (8–7)	−1.137	0.256*	0.20
	Pessimism	12.16 (11–6)	12.32 (12–6)	−0.242	0.809*	0.04
	Social Isolation/Mistrust	15.03 (13–10)	15.90 (14–12)	−1.938	0.053*	0.35
	Emotional Inhibition	10.00 (10–6)	10.55 (10–10)	−1.134	0.257*	0.20
	Approval-Seeking	17.39 (18–11)	18.45 (18–9)	−1.679	0.093*	0.30
	Enmeshment/Dependence	16.26 (12–10)	16.58 (14–8)	−0.898	0.369*	0.16
	Entitlement/Insufficient Self-Control	19.42 (18–13)	20.65 (20–16)	−2.736	0.006*	0.49
	Abandonment	8.87 (7–5)	10.16 (10–6)	−2.356	0.018*	0.42
	Punitiveness	18.35 (19–13)	18.23 (19–12)	−0.245	0.807*	0.04
	Defectiveness	10.74 (8–6)	11.26 (8–6)	−1.316	0.188*	0.24
	Vulnerability to Harm	10.35 (10–5)	10.55 (9–6)	−0.510	0.610*	0.09
	Unrelenting Standards	7.84 (6–8)	8.42 (7–8)	−1.990	0.047*	0.36
	Total	204.19 (190–112)	213.00 (195–104)	−2.870	0.004*	0.52

BDI: Beck Depression Inventory; QOLIE-31: Quality of Life in Epilepsy Inventory; YSQ-SF3: Young Schema Questionnaire–Short Form 3; IQR: interquartile range.

* This table presents variables that did not meet normal distribution assumptions and were analyzed using the Wilcoxon signed-rank test, including BDI scores.

**Table 3:** Correlations between PTS levels and clinical variables at month 1 and month 6.

		PCL-5			PCL-5		
		Month 1 (r)	P	FDR-adjusted q value	Month 6 (r)	P	FDR-adjusted q value
Age		−0.248	0.179	0.403	−0.529	0.002	0.010^*^
BDI		0.169	0.363	0.653	0.588	0.001	0.010^*^
BAI		0.472	0.007	0.189	0.703	<0.001	0.010^*^
QOLIE-31	Seizure Worry	−0.437	0.014	0.189	−0.364	0.044	0.149
	Emotional Wellbeing	0.046	0.807	0.860	0.110	0.555	0.849
	Energy Fatigue	−0.015	0.935	0.935	0.062	0.742	0.919
	Social Function	−0.041	0.827	0.860	−0.002	0.993	0.993
	Cognitive Function	−0.343	0.059	0.258	−0.421	0.018	0.078
	Medication Effect	0.122	0.515	0.732	−0.048	0.797	0.926
	Overall Quality of Life	−0.321	0.079	0.267	−0.075	0.689	0.896
	Overall Health Status	−0.357	0.049	0.258	−0.526	0.002	0.010^*^
	Total	−0.386	0.032	0.258	−0.532	0.002	0.010^*^
YSQ-SF3	Emotional Deprivation	0.123	0.509	0.732	−0.016	0.930	0.967
	Failure	0.155	0.405	0.683	0.138	0.460	0.797
	Pessimism	0.274	0.136	0.334	0.266	0.148	0.321
	Social Isolation/Mistrust	0.334	0.067	0.258	0.336	0.065	0.188
	Emotional Inhibition	0.077	0.681	0.860	0.172	0.354	0.708
	Approval-Seeking	0.047	0.802	0.860	−0.043	0.819	0.926
	Enmeshment/Dependence	0.355	0.050	0.258	0.310	0.089	0.231
	Entitlement/Insufficient Self-Control	0.052	0.780	0.860	0.085	0.649	0.896
	Self-Sacrifice	−0.233	0.208	0.432	0.140	0.453	0.797
	Abandonment	0.202	0.276	0.532	0.361	0.046	0.149
	Punitiveness	0.042	0.821	0.860	−0.032	0.865	0.937
	Defectiveness	0.041	0.828	0.860	0.290	0.114	0.269
	Vulnerability to Harm	0.301	0.099	0.292	0.076	0.684	0.896
	Unrelenting Standards	−0.294	0.108	0.292	−0.121	0.518	0.842
	Total	0.133	0.476	0.732	0.153	0.404	0.681

PCL-5: Post-Traumatic Stress Disorder Checklist for DSM-5; BDI: Beck Depression Inventory; BAI: Beck Anxiety Inventory; QOLIE-31: Quality of Life in Epilepsy Inventory; YSQ-SF3: Young Schema Questionnaire–Short Form 3.

**Table 4:** Multiple linear regression analyses predicting PCL-5 scores at month 1 and month 6.

Month 1					
Independent Variable	B (95%CI)	Std. Error	β (Beta)	t	p
(Constant)	13.087	5.652		2.316	0.029*
Sex	−11.726 (−19.79 to −3.67)	3.921	−0.366	−2.991	0.006*
BAI	0.447 (0.11 to 0.78)	0.163	0.348	2.743	0.011*
Presence of Physical Trauma in First Seizure	−8.616 (−16.69 to −0.55)	3.926	−0.276	−2.195	0.037*
YSQ-SF3 – Enmeshment/Dependence	0.772 (0.30 to 1.24)	0.229	0.424	3.377	0.002*
Month 6					
Independent Variable	B (95%CI)	Std. Error	β (Beta)	t	p
(Constant)	17.099	3.987		4.289	<0.001*
BAI	0.856 (0.51 to 1.21)	0.171	0.682	5.018	<0.001*

PCL-5: Post-Traumatic Stress Disorder Checklist for DSM-5; BAI: Beck Anxiety Inventory; YSQ-SF3: Young Schema Questionnaire–Short Form 3; B: unstandardized regression coefficient; Std. Error: standard error; β: standardized regression coefficient; CI: confidence interval;

* multiple linear regression analyses.

Model summary: Month 1 – F (4, 26) = 10.449, p < 0.001, R_2_ = 0.79, adjusted R_2_ = 0.56; Month 6 – F (1, 29) = 25.176, p < 0.001, R_2_ = 0.68, adjusted R_2_ = 0.45.

## Data Availability

All data have been anonymized and no participant information has been shared with third parties or institutions. The data were used solely for scientific analysis purposes. The data that support the findings of this study are available from the corresponding author upon reasonable request.
